# Polygenic Risk, Agent Orange Exposure, and Lymphoid Neoplasms in the Veterans Affairs Million Veteran Program

**DOI:** 10.1001/jamanetworkopen.2025.26787

**Published:** 2025-08-13

**Authors:** Xueyi Teng, Jia Y. Wan, Pankaj Gupta, Wei Li, Wendy Cozen, Helen Ma

**Affiliations:** 1 Department of Biological Chemistry, School of Medicine, University of California, Irvine; 2 Department of Epidemiology and Biostatistics, Joe C. Wen School of Population & Public Health, University of California, Irvine; 3 Tibor Rubin Veterans Affairs Medical Center, Long Beach, California; 4 Division of Hematology/Oncology, Department of Medicine, University of California, Irvine; 5 Chao Family Comprehensive Cancer Center, University of California, Irvine

## Abstract

**Importance:**

Agent Orange (AO), a toxic herbicide mixture contaminated with carcinogenic dioxin, was widely used during the Vietnam War. Although epidemiologic evidence suggests AO exposure is associated with increased lymphoid malignant neoplasm risk, the details of this association and its potential interaction with genetic factors remain unclear.

**Objective:**

To evaluate (1) whether AO exposure was associated with increased risk of lymphoid malignant neoplasms, (2) associations of polygenic risk scores (PRSs) in specific lymphoid malignant neoplasms, and (3) whether there was a genetic component × exposure interaction associated with increased risk of developing lymphoid malignant neoplasms.

**Design, Setting, and Participants:**

A case-control study of 255 155 US veterans enrolled in the Veterans Affairs Million Veteran Program was conducted with available genotype, AO exposure information, and lymphoid malignant neoplasm diagnosis from January 1, 1965, through June 11, 2024.

**Exposures:**

AO exposure status (based on self-reported survey) and PRS derived from genome-wide association studies of lymphoid malignant neoplasms.

**Main Outcomes and Measures:**

Risk of developing lymphoid malignant neoplasm subtypes: chronic lymphocytic leukemia, diffuse large B-cell lymphoma, follicular lymphoma, marginal zone lymphoma, and multiple myeloma.

**Results:**

A total of 255 155 non-Hispanic White veterans (median [IQR] age, 67 [61-74] years; 235 895 [92.5%] male) with both PRS and AO data were included in this study. AO exposure was associated with an increased risk of chronic lymphocytic leukemia (odds ratio [OR], 1.61; 95% CI, 1.40-1.84), diffuse large B-cell lymphoma (OR, 1.26; 95% CI, 1.03-1.53), follicular lymphoma (OR, 1.71; 95% CI, 1.39-2.11), and multiple myeloma (OR, 1.58; 95% CI, 1.35-1.86). Similarly, PRS was associated with all subtypes: chronic lymphocytic leukemia (OR, 1.81; 95% CI, 1.70-1.93), diffuse large B-cell lymphoma (OR, 1.12; 95% CI, 1.02-1.21), follicular lymphoma (OR, 1.33; 95% CI, 1.21-1.47), marginal zone lymphoma (OR, 1.17; 95% CI, 1.04-1.32), and multiple myeloma (OR, 1.41; 95% CI, 1.31-1.52). No significant PRS × AO interactions were observed.

**Conclusions and Relevance:**

To date, this is the largest case-control study of AO exposure and risk of lymphoid malignant neoplasm subtypes and the only study to investigate an AO × gene association with risk. The study found independent associations of both genetic predisposition and AO exposure on several lymphoid malignant neoplasm subtypes.

## Key Points

**Question:**

Is Agent Orange (AO) exposure associated with increased risk of developing mature lymphoid malignant neoplasms, and is there a joint association of AO exposure and polygenic risk?

**Findings:**

In this case-control study of 255 155 US veterans, both the polygenic risk score and AO exposure were associated with the development of chronic lymphocytic leukemia, diffuse large B-cell lymphoma, follicular lymphoma, and multiple myeloma. No significant polygenic risk score × AO interaction was observed in any of these lymphoid malignant neoplasms.

**Meaning:**

These findings suggest that AO exposure and polygenic risk independently contribute to lymphoma malignant neoplasm risk without evidence of interaction, pointing to distinct and potentially additive pathways.

## Teaser

This case-control study of veterans evaluates the associations of Agent Orange exposure with increased risk of and polygenic risk scores in specific lymphoid malignant neoplasms as well as whether there was a genetic component × exposure interaction associated with increased risk of developing lymphoid malignant tumors.

## Introduction

Non-Hodgkin lymphomas (NHLs) are the most common hematologic malignant neoplasms (2.9%) diagnosed in US veterans using the Veterans Affairs (VA) health care system.^1^ Male US Army and Marine Corps Vietnam veterans have increased mortality (proportional mortality ratio, 2.10; 95% CI, 1.17-3.79) from NHLs compared with non-Vietnam veterans.^2^ Exposures, including Agent Orange (AO), during the Vietnam War could put military personnel at higher risk of death from certain cancers. AO was a toxic herbicide mixture that was contaminated by 2,3,7,8-tetrachlorodibenzo-p-dioxin (TCDD), a carcinogenic byproduct.^3^ The World Health Organization International Agency for Research on Cancer concluded that there is sufficient evidence that TCDD is associated with increased risk of NHLs and other cancers based on human nested case-control and cohort studies as well as animal carcinogenicity data.^4^ TCDD exposure induces proliferation of hematopoietic stem cells as well as immune suppression, possibly leading to increased risk of lymphoid malignant neoplasms.^5^ Although the National Academies of Sciences, Engineering, and Medicine released a comprehensive analysis of epidemiologic studies that suggest there are sufficient data to support an association between AO and lymphoid malignant neoplasm risk, supportive studies related to AO exposure are small without analysis of specific subtypes.^3^

In addition to environmental agents, genome-wide association studies (GWASs) have revealed a genetic predisposition in the development of lymphoid malignant neoplasms. Most loci are concentrated in pathways related to immune regulation, apoptosis, and regulation of oncogene expression. For instance, human leukocyte antigen genes, vital in immune response and autoimmune disease, are associated with lymphoid malignant neoplasms.^6,7,8,9,10,11^ In addition, *BCL2* is a firmly established risk locus in multiple lymphomas and involved in apoptosis of lymphocytes.^8,12^ The genetic risk factors can be summed into a score that measures the degree of risk based on the number of individual risk variants that a person has. This score is called the polygenic risk score (PRS), with the higher scores indicating higher risk of disease.^13^ Although GWASs have identified common and unique susceptibility loci in subtypes of lymphoid malignant neoplasms, none are associated with all lymphoid malignant neoplasm subtypes, which highlights the heterogeneity of lymphomagenesis.^12^ Moreover, a limitation is that previous GWASs did not take environmental and other nongenetic exposures into account. Lymphomagenesis is likely due to a combination of several types of effectors.

The VA Million Veteran Program (MVP) is a large observational cohort study that combines surveys, electronic health records, and whole genome sequencing in the VA health care system.^14^ Access to the large lymphoid malignant neoplasm cohort in this unique database allows for the opportunity to evaluate for genetic and environmental associations in Vietnam War era veterans and the association with lymphoid malignant neoplasms. The objective of this research study was to evaluate (1) whether AO exposure was associated with increased risk of lymphoid malignant neoplasms, (2) associations of polygenic risk scores (PRSs) in specific lymphoid malignant neoplasms, and (c) whether there was a genetic component × exposure interaction associated with increased risk of developing lymphoid malignant neoplasms.

## Methods

### Case Definition

This study was approved by the VA Central Institutional Review Board and the local Research and Development Committee. A waiver of patient informed consent form was granted by the VA Central Institutional Review Board because this was a secondary analysis of coded data. This study followed the Strengthening the Reporting of Observational Studies in Epidemiology (STROBE) reporting guideline for case-control studies. This case-control study used data from the MVP cohort. Mature lymphoid malignant neoplasm cases were identified using the *International Classification of Diseases for Oncology*, *Third Edition (ICD-O-3)* from the VA Central Cancer Registry linked to MVP. Given the ancestry distribution of NHL cases with AO exposure data in the MVP cohort (403 African American, 172 Hispanic, and 2640 non-Hispanic White individuals), we restricted our analyses to ancestry groups for which GWASs were available (ie, non-Hispanic White veterans for all subtypes and non-Hispanic Black veterans for MM, to avoid potential confounding due to population structure and limited sample sizes in other ancestry groups). For this reason, data on race and ethnicity were reported. Race and ethnicity were self-reported in the MVP survey.^15^ The control group consisted of veterans without a diagnosis of lymphoid malignant neoplasms from January 1, 1965, through June 11, 2024 (date of data retrieval).

Because lymphoid malignant neoplasms may be secondary cancers and have the rare potential to transform to another type, we considered the initial lymphoid malignant neoplasm diagnosis as the primary diagnosis for each patient. For example, the case group of chronic lymphocytic leukemia (CLL) included the patients whose first lymphoid malignant neoplasm diagnosis was CLL. The following lymphoid malignant neoplasms that had published PRS data were considered as the initial diagnosis and included in the analysis: CLL, diffuse large B-cell lymphoma (DLBCL), follicular lymphoma (FL), marginal zone lymphoma (MZL), and multiple myeloma (MM). AO exposure was defined by self-report in the MVP baseline survey by answering “yes,” “no,” or “not sure” to the question, “Were you ever exposed to Agent Orange?”

### Genotype Data

MVP genotype data were generated by customized Affymetrix Axiom biobank array (MVP, release 3) and imputed with the 1000 Genome Project reference panel.^14,16^ Variant and sample-level quality control were performed according to the MVP protocol as previously published.^17^ Ancestry-specific Hardy-Weinberg equilibrium *P* < 1^−20^, posterior call probability less than 0.9, imputation quality/INFO less than 0.3, minor allele frequency less than 0.0003, call rate less than 97.5% for common variants (minor allele frequency >1%), and call rate less than 99% for rare variants (minor allele frequency <1%) were used.^18^ Variants were also excluded if they deviated by more than 10% from their expected allele frequency based on reference data from the 1000 Genome Project.^19^ Genetic principal components (PCs) were supplied by MVP.

### Patient Selection

We started with 317 126 non-Hispanic White individuals in our cohort (3268 with a lymphoid malignant neoplasm and 313 858 controls). A total of 628 samples in all lymphoid malignant neoplasm groups (214 with CLLs, 131 with DLBCLs, 101 with FLs, 41 with MZLs, and 141 with MMs) and 61 343 samples in control groups were excluded due to the unavailability of AO exposure information (ie, not available or unknown). For the MM cohort, we also excluded 360 samples with monoclonal gammopathy (a precursor of MM) from the control group. The final cohort included a total of 255 155 individuals.

For the analysis of MM in African American individuals, we selected non-Hispanic African American individuals from the MVP cohort using the same inclusion criteria applied to the non-Hispanic White group. The MM cases were required to have MM as their initial lymphoid malignant neoplasm diagnosis, and individuals in the control group must not have had a monoclonal gammopathy (or MM) diagnosis.

### Statistical Analysis

#### Main Analysis 

Lymphoid malignant neoplasm risk single-nucleotide variants (SNVs) and odds ratios (OR), used for polygenic score calculation, were obtained from previously published GWASs listed in eTable 1 in Supplement 1.^12,20^ For the African American MM models, we applied the PRS reported in a previous study^21^ of an MM GWAS among African American patients and controls. The Wilcoxon rank sum test was then used to compare PRSs in the cancer and control groups. We used β*_i_* = ln (OR*_i_*) weights for each i-th SNV in the PRS calculation with plink, version 1.9.^22^ The PRS was calculated as a linear combination of SNV additive genotypes (G*_i_* coded 0, 1, 2 copies of the effect allele) and corresponding weights β*_i_*, such that PRS =  ∑ *_i_* _ = 1_*^N^*_SNV_β*_i_* × *G_i_* where *i* is 1 to the number of SNVs in the PRS. PRSs were standardized by subtracting the mean and then dividing by the SD, resulting in a standardized PRS score with a mean (SD) of 0 (1).

In our logistic regression models, we adjusted for PRSs and AO exposure in 2 separate models, both adjusting for the following confounders: age at MVP enrollment, sex, and the first 10 genetic PCs.^14^ The formula for the PRS model is log (*i*) *t*(*p*) = β_0_ + β_PRS_PRS + β_age_age + β_sex_sex + β_PC1_ + … + βP_C10_PC10.

The formula for the AO model is log (*i*) *t*(*p*) = β_0_ + β_AO_*E*_AO_ + β_age_age + β_sex_sex + β_PC1_ + … + βP_C10_PC10.

We then established the following model to assess the associations between PRS × AO and lymphoid malignant neoplasm development using multivariable unconditional logistic regression. The formula for the PRS × AO interaction model is log (*i*) *t*(*p*) = β_0_ + β_PRS_PRS+ β_AO_*E*_AO_ + γ_PRS × AO_PRS × *E*_AO_ + β_age_age + β_sex_sex + β_PC1_ + … + βP_C10_PC10. Here, p represents the probability of the phenotype (1 indicates specific lymphoid malignant neoplasms and 0 indicates control), E_AO_ signifies the AO exposure (1 indicates exposed and 0 indicates unexposed environmental component). The term PRS × E_AO_ indicates polygenic × environment interaction. Age at MVP enrollment, sex, and the top 10 PCs (PC1, PC2, …PC10) were included as covariates. The model was fitted using logistic regression, and parameter coefficients were estimated. The Wald test was used to determine whether there was a significant risk of lymphoid malignant neoplasms associated with PRS, AO, and the interaction of PRS × AO. Two-sided *P* < .05 was considered statistically significant. To further assess the PRS × AO interaction, we evaluated the ORs and 95% CIs of having lymphoid malignant neoplasms for each SD increase in PRS within subsets of each AO-exposed and -unexposed groups. All statistical analyses were performed with R software, version 4.2.2 and PLINK, version 1.9.^22^

#### Sensitivity Analyses

We conducted sensitivity analyses in 2 separate subcohorts. To address potential bias due to the limited number of female study participants, we restricted the analysis to male participants. We also assessed potential recall bias by restricting the analysis to incident cases, defined as individuals whose age at cancer diagnosis was older than their age at MVP enrollment.

## Results

### Patient Characteristics

After filtering samples with both genotype and AO exposure information, we included 255 155 non-Hispanic White participants (median [IQR] age, 67 [61-74] years; 235 895 [92.5%] male and 19 260 [7.5%] female) in the study, comprising of 70 037 AO-exposed and 185 118 AO-unexposed individuals (Table 1). There were 2640 cases of 5 different lymphoid malignant neoplasms, including CLL, DLBCL, FL, MZL, and MM, among which 973 individuals were exposed to AO according to their clinical records. Because only 6 cases (0.2%) were diagnosed between January 1965 and May 1975, nearly all cases of lymphoid malignant neoplasms were diagnosed after the period of AO exposure risk.

**Table 1.** Agent Orange–Exposed and Unexposed Veterans by Baseline Characteristics in the MVP Cohort

Characteristic	Exposed (n = 70 037)	Unexposed (n = 185 118)	All (N = 255 155)
Age at MVP enrollment, median (IQR), y	68 (65-70)	67 (57-77)	67 (61-74)
Age at diagnosis, median (IQR), y^a^			
CLL	67 (62-71)	73 (65-80)	70 (63-77)
DLBCL	68 (65-73)	69 (60-77)	69 (62-75)
FL	66 (61-72)	69 (61-77)	67 (61-74)
MZL	67 (62-71)	72 (62-82)	69 (62-78)
MM	68 (64-73)	73 (66-80)	70 (65-77)
Sex, No. (%)			
Male	69 700 (99.5)	166 195 (89.8)	235 895 (92.5)
Female	337 (0.5)	18 923 (10.2)	19 260 (7.5)
Lymphoid malignant neoplasms, No.^b^	973	1667	2640
Lymphoid malignant neoplasm subtype, No. (%)			
CLL	358 (36.8)	586 (35.2)	944 (35.8)
DLBCL	155 (16.0)	314 (18.8)	469 (17.8)
FL	150 (15.4)	228 (13.7)	378 (14.3)
MZL	58 (6.0)	138 (8.3)	196 (7.4)
MM	252 (25.9)	401 (24.1)	653 (24.7)
Controls, No.	69 064	183 451	252 515

Abbreviations: CLL, chronic lymphocytic leukemia; DLBCL, diffuse large B-cell lymphoma; FL, follicular lymphoma; MM, multiple myeloma; MVP, Million Veteran Program; MZL, marginal zone lymphoma.

^a^ Only included participants diagnosed with lymphoid malignant neoplasms.

^b^ Lymphoid malignant neoplasms categorized as not otherwise specified were omitted.

### Separate Associations of PRS and AO Adjusted for Confounders

**Figure 1.**
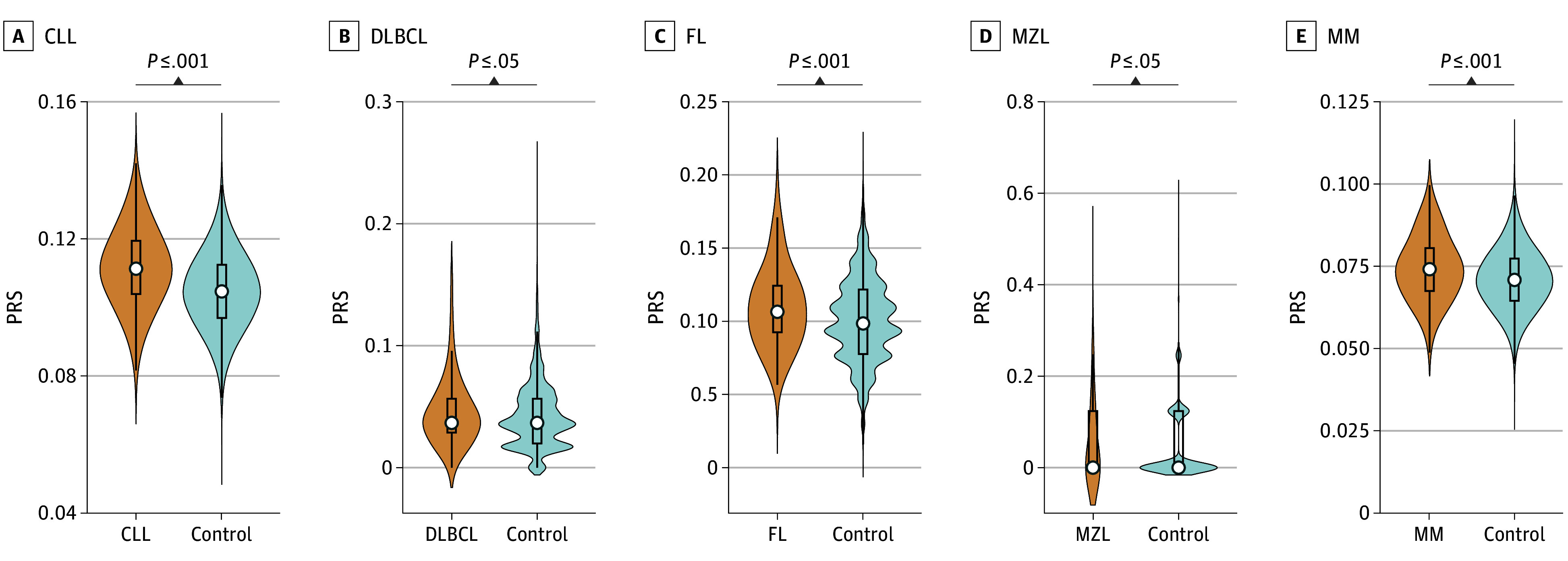
Polygenic Risk Scores (PRSs) in Subtypes of Lymphoid Malignant Neoplasms Compared With Controls Mean test between lymphoid malignant neoplasms and control group was produced by the Wilcoxon rank sum test. CLL indicates chronic lymphocytic leukemia; DLBCL, diffuse large B-cell lymphoma; FL, follicular lymphoma; MM, multiple myeloma; MZL, marginal zone lymphoma.

PRSs were higher in cases than in controls across all subtypes of mature B-cell lymphoma in our cohort (Figure 1), with the largest differences observed for CLL, FL, and MM. AO exposure was associated with the risk of certain subtypes of mature B-lymphoid malignant neoplasms (OR, 1.61 [95% CI, 1.40-1.84] for CLL; OR, 1.26 [95% CI, 1.03-1.53] for DLBCL; OR, 1.71 [95% CI, 1.39-2.11] for FL; and OR, 1.58 [95% CI, 1.35-1.86] for MM) (Table 2; eTable 2 in Supplement 1). The association of PRS on lymphoid malignant neoplasm risk is detailed in Table 2 (eTable 3 in 1 Supplement 1). The highest OR for association with AO exposure was for FL (OR, 1.71; 95% CI, 1.39-2.11), whereas the highest OR for association with PRS was for CLL (OR, 1.81; 95% CI, 1.70-1.93).

**Table 2.** Association Models for Separate Associations of Agent Orange and Polygenic Risk Score Exposure With Development of Lymphoid Malignant Neoplasms

Lymphoid malignant neoplasm subtype^a^	AO exposure^b^		PRS^c^	
	OR (95% CI)	*P* value	OR (95% CI)	*P* value
CLL	1.61 (1.40-1.84)	<.001^d^	1.81 (1.70-1.93)	<.001^d^
DLBCL	1.26 (1.03-1.53)	.02^d^	1.12 (1.02-1.21)	.01^d^
FL	1.71 (1.39-2.11)	<.001^d^	1.33 (1.21-1.47)	<.001^d^
MZL	1.11 (0.81-1.52)	.51	1.17 (1.04-1.32)	.01^d^
MM	1.58 (1.35-1.86)	<.001^d^	1.41 (1.31-1.52)	<.001^d^

Abbreviations: AO, Agent Orange; CLL, chronic lymphocytic leukemia; DLBCL, diffuse large B-cell lymphoma; FL, follicular lymphoma; MM, multiple myeloma; MZL, marginal zone lymphoma; OR, odds ratio; PRS, polygenic risk score.

^a^ Lymphoid malignant neoplasm subtype vs controls.

^b^ Logistic regression adjusted for Agent Orange exposure, age, sex, and the first 10 genetic principal components.

^c^ Logistic regression adjusted for polygenic risk score, age, sex, and the first 10 genetic principal components.

^d^ Statistically significant.

Given that MM had a comparatively larger number of African American cases (n = 230) and controls (n = 38 230) in the MVP cohort (eTable 5 in Supplement 1), we conducted a parallel analysis within the African American group to assess the consistency of AO and PRS associations with MM risk across ancestries. Both AO exposure (OR, 1.56; 95% CI, 1.18-2.07) and PRS (OR, 1.31; 95% CI, 1.15-1.49) were associated with MM risk in African American participants, mirroring the findings observed in non-Hispanic White participants (eTable 5 in Supplement 1).

### Interaction of PRS Genetic Component and AO Exposure in Lymphoid Malignant Neoplasm Risk

**Figure 2.**
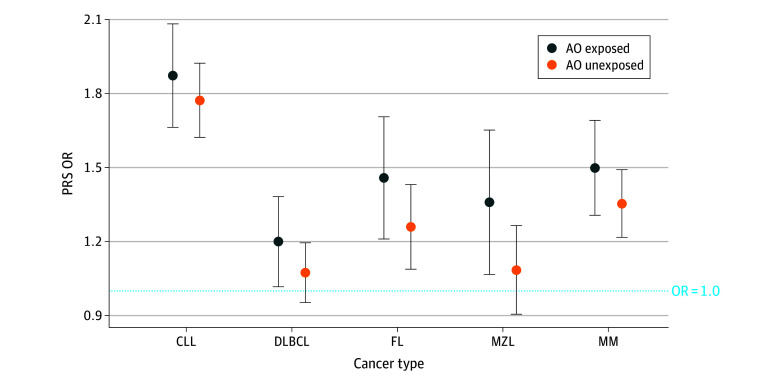
Association of Polygenic Risk Score (PRS) With Lymphoid Malignant Neoplasm Development in Agent Orange (AO)–Exposed Samples Error bars indicate 95% CIs. CLL indicates chronic lymphocytic leukemia; DLBCL, diffuse large B-cell lymphoma; FL, follicular lymphoma; MM, multiple myeloma; MZL, marginal zone lymphoma; OR, odds ratio.

Although there were independent associations of PRS and AO exposure, there was no PRS and AO interaction in the risk of any of the lymphoid malignant neoplasm subtypes (Table 3; eTable 4 in Supplement 1). Parallel analysis in African American MM cases similarly did not reveal any interaction between PRS and AO exposure (eTable 5 in Supplement 1). However, when we compared the OR of the PRS genetic term of the AO-exposed group with that of the AO-unexposed group, we found that the OR was higher in the AO-exposed group in all subtypes of lymphoid malignant neoplasms but the differences were not statistically significant (CLL, DLBCL, FL, MZL, and MM) (Figure 2).

**Table 3.** Joint Associations of PRS, AO Exposure, and Interaction by Lymphoid Malignant Neoplasm Subtype

Lymphoid malignant neoplasm subtype^a^	PRS		AO exposure		PRS × AO^b^	
	OR (95% CI)	*P* value	OR (95% CI)	*P* value	ROR (95% CI)^c^	*P* value
CLL	1.77 (1.63-1.92)	<.001^d^	1.55 (1.33-1.82)	<.001^d^	1.06 (0.93-1.20)	.41
DLBCL	1.07 (0.96-1.19)	.19	1.24 (1.02-1.51)	.03^d^	1.12 (0.94-1.34)	.22
FL	1.26 (1.11-1.43)	<.001^d^	1.63 (1.31-2.03)	<.001^d^	1.16 (0.95-1.42)	.14
MZL	1.09 (0.93-1.26)	.28	1.05 (0.76-1.45)	.75	1.25 (0.98-1.60)	.08
MM	1.36 (1.23-1.49)	<.001^d^	1.53 (1.29-1.81)	<.001^d^	1.10 (0.95-1.29)	.21

Abbreviations: AO, Agent Orange; CLL, chronic lymphocytic leukemia; DLBCL, diffuse large B-cell lymphoma; FL, follicular lymphoma; MM, multiple myeloma; MZL, marginal zone lymphoma; OR, odds ratio; PRS, polygenic risk score; ROR, ratio of odds ratios.

^a^ Lymphoid malignant neoplasm subtype vs controls.

^b^ Using logistic regression interaction, PRS × AO model adjusted for age, sex, and the first 10 genetic principal components.

^c^ The ratio of the PRS OR in the AO-exposed category divided by the PRS OR in the AO-unexposed category or those whose value of the risk factor is 1 unit less.

^d^ Statistically significant.

## Discussion

We investigated the association among AO exposure, genetic risk, and the interaction of the 2 on the risk of developing lymphoid malignant neoplasm subtypes. Our results show AO exposure was associated with the risk of developing CLL, DLBCL, FL, and MM. Polygenic risk and development of a lymphoid malignant neoplasm were associated in all subtypes, consistent with previous studies.^8,9,10,20,23^

A previous population-based study^24^ of 1157 Vietnam veterans compared with 1776 controls reported that there was an increased risk of development of NHLs in Vietnam veterans (OR, 1.47). However, the risk of specific lymphoma subtypes was not reported. Previous work^25^ comparing outcomes of AO exposure in subtypes of lymphoid malignant neoplasms did not evaluate the association of AO exposure and risk of developing cancer. Lymphomas are a highly heterogeneous group of cancers, and subtypes are associated with occupational exposures, infections, and immune dysregulation.^26^ A large case-control analysis of 17 471 cases and 23 096 controls from the International Lymphoma Epidemiology Consortium showed that general farm workers had an increased risk of NHL with an OR of 1.28 (95% CI, 1.10-1.50).^26,27^ This association was also seen in specific subtypes, such as CLL, suggesting an association of these subtypes of NHL with environmental exposures found in farming, such as herbicides. Our study not only supports that exposure to AO, a dioxin-contaminated herbicide, is associated with increased risk of subtypes of lymphoid malignant neoplasms but also is one of the first to quantify the ORs between AO exposure and the risk of developing CLL, DLBCL, FL, MZL, and MM. Dioxin is a known carcinogen, and one of its mechanisms is through immunosuppression, which is a risk factor in the development of lymphoid malignant neoplasms.^5^

Chemical carcinogens such as organochlorines have been shown to have potential interactions with genotype in lymphomagenesis.^28^ Studies^5,29^ have shown that TCDD can affect carcinogenesis progression through pathways of apoptosis and immune suppression, where risk loci for lymphoid malignant neoplasms are enriched. However, we did not find a significant interaction among PRS, AO exposure, and the development of lymphoid malignant neoplasm subtypes.

### Strengths and Limitations

The strengths of our study include (1) the large veteran cohort with information on AO exposure, (2) well-annotated clinical and genetic data, and (3) long-term follow-up (starting from the AO exposure onset in January 1962 to June 2024). However, the study also has some limitations. Although this is, to our knowledge, the largest study of AO exposure and genetic risk in lymphoid malignant neoplasm development, the power to find interaction associations in specific subtypes might be limited. Self-reported AO exposure may lead to survival bias, especially in aggressive subtypes, as well as recall bias. In addition, it is possible that patients with aggressive tumors died and were not at risk of joining the MVP. In our cohort, approximately half of the patients were diagnosed with lymphoid malignant neoplasm before self-reporting of AO exposure in the MVP survey. These individuals may have been more likely to recall or report prior AO exposure after being diagnosed with cancer (recall bias). To address potential recall bias, we performed a sensitivity analysis restricted to cases of lymphoid malignant neoplasms diagnosed after enrollment on MVP. The results of the sensitivity analysis were mostly consistent with those from the full cohort, suggesting that recall bias was unlikely to be a factor in the observed associations (eTable 6 in Supplement 1). Although only the OR of MZL was no longer significant in incident cases, this may be partly due to the small sample size. Likewise, given the difference in the proportion of female participants in the AO-exposed and AO-unexposed groups, we performed an additional sensitivity analysis with only male individuals. The results were also consistent with those of the primary analysis, further supporting that the observed associations were not biased by sex-related differences. If it had been available, quantifying AO exposure to include duration may have minimized the heterogeneity of AO exposure rather than with a dichotomous assessment as used in our current study. The MVP phenotype data do not include information on the exact timing of AO exposure, which limited our ability to assess age-specific risk patterns. Given the 1-year difference in median age at MVP enrollment between AO-exposed and AO-unexposed groups, some residual confounding by age may still have been present. PRSs have been calculated using data available from individuals of European descent because most genome association studies have been performed in individuals of European descent. Those studies that focus on diverse populations can contribute to our understanding of disease risk in different populations because the higher-risk variants in European descent may not apply to those with African descent in subtypes of lymphoid malignant neoplasms.^21^ The MVP cohort consists of a diverse population. If more GWASs from diverse populations were available, analyses on non-European populations would have contributed further to the knowledge base.

## Conclusions

In this case-control study of non-Hispanic White patients from the MVP, AO exposure and PRS had independent associations and higher ORs with the risk of developing CLL, DLBCL, FL, and MM. Our study addressed the public health concerns surrounding AO exposure and lymphoid malignant neoplasms, finding that both AO exposure and polygenic risk are independently associated with disease, suggesting potentially distinct and additive pathways that merit further investigation.

## Notes

Supplement. **eTable 1.** Single Nucleotide Polymorphisms (SNPs) in Polygenic Risk Score (PRS) Analysis **eTable 2.** Coefficients From Logistic Regression Estimating the Direct Association of AO Exposure **eTable 3.** Coefficients From Logistic Regression Estimating the Direct Association of PRS **eTable 4.** Coefficients From Logistic Regression Estimating PRS x AO Interactions **eTable 5.** Multiple Myeloma (MM) Risk Models in African Americans **eTable 6.** Sensitivity Tests

Supplement 2.
MVP Core Acknowledgment List

Supplement 3.
Data Sharing Statement
