# Error in Figure

**DOI:** 10.1001/jamanetworkopen.2025.38489

**Published:** 2025-09-16

In the Original Investigation titled “Polygenic Risk, Agent Orange Exposure, and Lymphoid Neoplasms in the Veterans Affairs Million Veteran Program,”^1^ published August 13, 2025, there was an error in Figure 1. In panel A, the label given twice as “CCL” should have been “CLL.” This article has been corrected.^1^
