# Supplementary material for: Polygenic Risk, Agent Orange Exposure, and Lymphoid Neoplasms in the Veterans Affairs Million Veteran Program
Source: JAMA Netw Open. 2025 Aug 13;8(8):e2526787. doi: 10.1001/jamanetworkopen.2025.26787 (PMC12351410; doi:10.1001/jamanetworkopen.2025.26787)
Supplement: Supplement 3. — Data Sharing Statement [file jamanetwopen-e2526787-s003.pdf]

Data Sharing Statement

Teng. Polygenic Risk, Agent Orange Exposure, and Lymphoid Neoplasms in the Veterans Affairs Million Veteran Program. *JAMA Netw Open*. Published August 13, 2025. doi:10.1001/jamanetworkopen.2025.26787

Data
Data available: No

Additional Information
Explanation for why data not available: We are not permitted to share data due to the sensitive nature of veteran data.
